# An observational study of workflows to support fecal testing for colorectal cancer screening in primary care practices serving Medicaid enrollees

**DOI:** 10.1186/s12885-021-09106-7

**Published:** 2022-01-25

**Authors:** Cynthia M. Mojica, Rose Gunn, Robyn Pham, Edward J. Miech, Ann Romer, Stephanie Renfro, Khaya D. Clark, Melinda M. Davis

**Affiliations:** 1 School of Social and Behavioral Health Sciences, College of Public Health and Human Sciences, Oregon State University, 2250 SW Jefferson Way, Corvallis, OR 97331 USA; 2 Oregon Rural Practice-based Research Network, Oregon Health & Science University, 3030 SW Moody Ave, Portland, OR 97201 USA; 3 Center for Health Services Research, Regenstrief Institute, 1101 W 10th St, Indianapolis, IN 46202 USA; 4 Center for Health Systems Effectiveness, Oregon Health & Science University, 3030 SW Moody Ave, Portland, OR 9720 USA; 5 Department of Medical Informatics and Clinical Epidemiology, Oregon Health & Science University, 3181 SW Sam Jackson Park Road, Portland, OR 97239 USA; 6 Department of Family Medicine and School of Public Health, Oregon Rural Practice-based Research Network, Oregon Health & Science University, 3030 SW Moody Ave, Portland, OR 97201 USA

**Keywords:** colorectal cancer, early detection of cancer, population health, primary health care, underserved populations, workflow

## Abstract

**Abstract:**

**Background:**

Screening supports early detection and treatment of colorectal cancer (CRC). Provision of fecal immunochemical tests/fecal occult blood tests (FIT/FOBT) in primary care can increase CRC screening, particularly in populations experiencing health disparities. This study was conducted to describe clinical workflows for FIT/FOBT in Oregon primary care practices and to identify specific workflow processes that might be associated (alone or in combination) with higher (versus lower) CRC screening rates.

**Methods:**

Primary care practices were rank ordered by CRC screening rates in Oregon Medicaid enrollees who turned age 50 years from January 2013 to June 2014 (i.e., newly age-eligible). Practices were recruited via purposive sampling based on organizational characteristics and CRC screening rates. Data collected were from surveys, observation visits, and informal interviews, and used to create practice-level CRC screening workflow reports. Data were analyzed using descriptive statistics, qualitative data analysis using an immersion-crystallization process, and a matrix analysis approach.

**Results:**

All participating primary care practices (N=9) used visit-based workflows, and four higher performing and two lower performing used population outreach workflows to deliver FIT/FOBTs. However, higher performing practices (n=5) had more established workflows and staff to support activities. Visit-based strategies in higher performing practices included having dedicated staff identify patients due for CRC screening and training medical assistants to review FIT/FOBT instructions with patients. Population outreach strategies included having clinic staff generate lists and check them for accuracy prior to direct mailing of kits to patients. For both workflow types, higher performing clinics routinely utilized systems for patient reminders and follow-up after FIT/FOBT distribution.

**Conclusions:**

Primary care practices with higher CRC screening rates among newly age-eligible Medicaid enrollees had more established visit-based and population outreach workflows to support identifying patients due for screening, FIT/FOBT distribution, reminders, and follow up. Key to practices with higher CRC screening was having medical assistants discuss and review FIT/FOBT screening and instructions with patients. Findings present important workflow processes for primary care practices and may facilitate the implementation of evidence-based interventions into real-world, clinical settings.

## Background

Colorectal cancer (CRC) usually begins as a noncancerous growth (i.e., polyp) that develops on the inner lining of the colon or rectum and grows slowly, over a period of 10 to 20 years [1]. When CRC is detected at an early localized-stage, the five-year survival rate is 90% compared to 14% when diagnosed at an advanced distant-stage [1]. CRC is preventable and treatable with guideline concordant screening [2]. Yet only 67% of age-eligible Americans are up-to-date with CRC screening [3, 4], which is well below national targets [5]. Low screening rates contribute to the fact that CRC remains the second leading cause of cancer death in the United States [6]. Moreover, persistent disparities exist in populations that are rural, ethnically diverse, and insured by Medicaid [7–10]. CRC screening in patients aged 50-64 years are also significantly lower than in patients over age 65 years [11]. Newly age-eligible Medicaid enrollees display especially low CRC screening rates with only 17% initiating screening within the first year after turning age 50 years [12] and 34.9% within 4 years of turning age 50 years [10].

The United States Preventive Services Task Force (USPSTF) and the American Cancer Society (ACS) recommend multiple modalities for CRC screening in average risk adults: [4, 13]: fecal immunochemical test or high-sensitivity fecal occult blood test every year, flexible sigmoidoscopy or CT colonography every 5 years, or colonoscopy every 10 years. At the time of this study, the USPSTF recommended individuals start screening at age 50 years whereas the ACS, in 2018, recommended starting at age 45 years. Colonoscopy is the most prevalent screening modality in the US, yet the procedure is expensive, invasive, and requires specialty medical providers [14]. Sigmoidoscopy was common before 2000, but has since been replaced by colonoscopy and comprises 3% of all CRC screenings [4]. Fecal testing using simple at-home fecal immunochemical tests (FIT) or high-sensitivity fecal occult blood tests (FOBT) to check for blood in the stool represents an important alternative modality for CRC screening based on cost, clinical effectiveness, and patient preference [15]. Many patients, specifically those in populations experiencing lower rates of CRC screening, prefer FIT/FOBT to colonoscopy [16]. However, fecal testing currently makes up less than 10% of all CRC screenings in the United States [1, 17].

An increasing body of literature highlights the important role of primary care in improving CRC screening rates by encouraging FIT/FOBT or referring patients for colonoscopy [10, 18]. To increase CRC screening, the Guide to Community Preventive Services encourages implementation of multicomponent, evidence-based interventions that use two or more strategies designed to increase community demand, community access, or provider delivery of screening services [19]. Although numerous studies have supported implementation of evidence-based interventions to increase CRC screening in primary care clinics [15, 18, 20–23], few studies describe clinical workflows for CRC screening in real-world primary care clinics. Clinical workflows are a series of physical and mental tasks performed by clinicians and staff within primary care practices or between care settings [24]. Implementing quality workflows are a vital step in facilitating the delivery of cancer screenings in primary care and ensuring appropriate clinical follow-up [25, 26].

This study, therefore, was conducted to describe clinical workflows for FIT/FOBT and to identify specific workflow processes that might be associated (alone or in combination) with higher (versus lower) CRC screening rates among Oregon primary care practices.

## Methods

This study used a sequential explanatory mixed methods design [27–30] informed by the positive deviance framework [31]. Data were collected from September 2016 to April 2017 as part of a larger study examining individual- and practice-level characteristics of CRC screening and screening modality among Medicaid enrollees in Oregon [12, 32]. The positive deviance framework includes four steps: [1] identifying practices demonstrating higher CRC screening rates, [2] conducting in-depth qualitative analysis to generate hypotheses about practices achieving higher CRC screening rates, [3] testing hypotheses in larger, representative samples, and [4] working with stakeholders to disseminate evidence regarding best practices [31]. This manuscript reports on the first two steps of the framework. Approval to conduct the study was received from the Oregon Health & Science University Institutional Review Board (IRB # 15847), with a full waiver of the HIPAA Authorization of written consent.

### Primary care practice sampling

Primary care practices were rank-ordered based on CRC screening rates calculated from Oregon Medicaid claims data. CRC screening rates were based on newly age-eligible Medicaid enrollees turning age 50 years between January 1, 2013 and June 30, 2015, and having received a colonoscopy, FIT/FOBT, or sigmoidoscopy in the year after turning age 50 years. The focus on newly age-eligible Medicaid enrollees allowed us to explore CRC screening initiation and eliminated challenges in exploring CRC screening up-to-date status given we did not have access to 10 years of claims data.

**Fig. 1:**
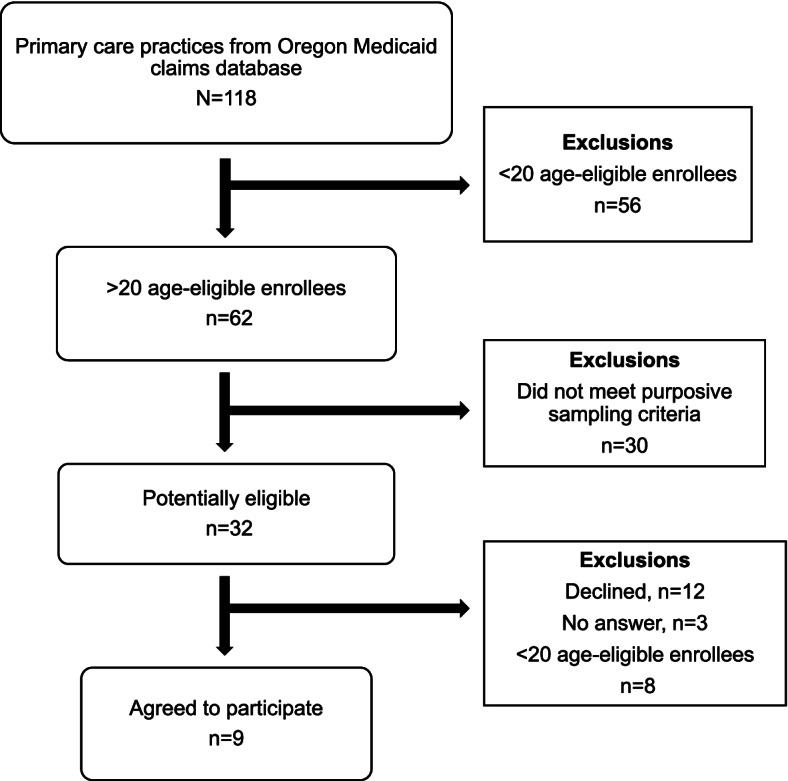
Primary care practice sampling

Figure 1 summarizes how the study sample was identified and recruited. First, practices (N=118) were rank ordered based on screening rate (range: 0% to 53.8%), and then practices with less than 20 newly age-eligible Medicaid enrollees (n=56) were excluded. Among the 62 remaining primary care practices, screening rates ranged from 3.4% to 51.9%. Next, purposive sampling based on key characteristics (e.g., ownership, geographic location, affiliation, and size) was used to identify 32 potentially eligible primary care practices. Study team members identified and approached the lead clinician, office manager, or other designated point of contact at each potentially eligible practice by sending recruitment emails and making follow-up phone calls. Nine practices (screening rates: 4.8% to 42.9%) agreed to participate, whereas 12 declined (screening rates: 3.4% to 51.9%); three did not respond (screening rates: 11.0%, 43.0%, and 46.0%) and eight (upon further review) did not meet the eligibility criteria of 20+ newly age-eligible Medicaid enrollees. Among practices that agreed to participate, practices with 32.9% to 42.9% screening rates were categorized as having “higher” CRC screening whereas practices with a 4.8% to 15.4% screening rate were categorized as having “lower” CRC screening. The higher versus lower designation was based on prior work in this population showing that only 17% of Medicaid enrollees are screened within the first year of turning age 50 years [12].

### Data collection

Data from each of the nine participating primary care practices were obtained from a baseline survey, observation visits, and informal interviews, and were used to create practice-level CRC screening workflow reports. The baseline survey was a 13-item questionnaire distributed prior to the observation visit that gathered information on the following practice characteristics: setting (urban versus rural), ownership (private, nonprofit, federally qualified health center, rural health clinic), affiliation (hospital, public health, health system), electronic health record (EHR) system used, number of medical clinicians (i.e., doctors of medicine or osteopathic medicine, nurse practitioners, and physician assistants), clinician FTE, and composition of patient insurance coverage.

The observation visits were conducted over two days by two research team members. Prior to the visits, research team members discussed (via a phone call) study goals and visit expectations (e.g., timing and logistics) with practice managers. During the visits, research team members observed patient pathways and shadowed clinical staff during patient preparation, medical exams, and follow-up visits. Brief informal interviews on the day of the observation visits also were conducted with clinical staff to clarify observations. Study team members took brief notes while observing and expanded these into typed, detailed field notes within 24 hours of each visit. Within two weeks of the observation visits, a workflow report for each practice was developed that included a brief summary of how data were collected during the visit, a description of observed CRC screening workflows using fecal testing and/or colonoscopy, and recommendations for improvement. Each practice-level workflow report was shared electronically with the practice manager and discussed with the practice manager and other clinical team members during a 45- to 60-minute phone call.

### Mixed methods analysis

The multidisciplinary team used a series of immersion crystallization cycles [33] to describe workflows for CRC testing in practices with higher versus lower CRC screening and matrix analysis to evaluate the degree to which specific workflow processes were associated with higher versus lower CRC screening [34, 35]. Field notes and workflow diagrams were entered into ATLAS.ti for data management and analysis.

The immersion-crystallization process involves immersion (i.e., examining portions of the data in detail) and crystallization (i.e., reflecting on the analysis and identifying patterns) [33]. In the first cycle, team members focused on describing key workflow steps, that is, activities to support CRC screening that occur within primary care practices (i.e., visit-based workflow) and outside of a scheduled appointment (i.e., population outreach workflows). In the second cycle, specific workflow processes for both visit-based and population outreach workflows were identified and focused on FIT/FOBT, as this was where practices displayed the greatest heterogeneity. In the third cycle, workflow activities and processes were compared across primary care practices to identify best practices.

The matrix analysis [34, 35] was used in this step to evaluate the degree to which specific workflow processes were associated with higher versus lower CRC screening. This approach involves coding field notes for the presence or absence of specific workflow processes and specifying an outcome. Two team members independently coded field notes and the outcome was defined as dichotomous and coded as 1 for practices with higher CRC screening and 0 for practices with lower CRC screening. A matrix display was generated by stratifying the data by the outcome and observing across each workflow process to identify which correspond to the presence of the outcome (i.e., higher CRC screening).

## Results

### Practice characteristics

Characteristics of the nine primary care practices appear in Table 1. Practice 1 to 5 had screening rates between 33% and 43%, and thus categorized as higher performing, whereas practice 6 to 9 had screening rates between 5% and 15%, and categorized as lower performing. Practice 2, 3, 4, and 5 are federally qualified health centers, practice 6 is a rural health clinic, and the remaining practices are private, non-profit entities. All practices use the Epic EHR; three different versions of Epic are used. The number of clinicians per practice varied from 5 to 34, with practice 3 not reporting this data. Among practices that reported patient insurance coverage, 20-52% of patients were covered by Medicaid.

**Table 1:** Descriptive characteristics of participating primary care practices (N=9), September 2016- April 2017

	Primary care practice								
	Higher Performing					Lower Performing			
	1 ^**a**^	2 ^**b**^	3 ^**b**^	4 ^**b**^	5 ^**b**^	6	7	8 ^**a**^	9 ^**b**^
**General Characteristics**									
CRC Screening Observed (%)	42.9	38.1	37.0	35.3	32.9	15.4	11.5	5.0	4.8
**Organizational Features**									
Setting	Urban	Rural	Urban	Urban	Urban	Rural	Rural	Urban	Urban
Ownership	Private, non-profit	FQHC	FQHC	FQHC	FQHC	Rural Health Clinic	Private, non-profit	Private, non-profit	Private, non-profit
Affiliation	Hospital	Public Health	System	System	Hospital	Hospital	Hospital	Hospital	Hospital
EHR System	Epic 2015	OCHIN Epic 2014	Epic 2015	Epic 2015	OCHIN Epic 2014	OCHIN Epic 2014	Epic 2015	Epic 2015	Epic 2014
Medical clinicians ^**c**^	12	5	8	11	34	22	6	7	12
Clinician FTE	9.1	4.3	*	6.45	20.02	15.0	6.0	6.3	8.35
**Patient Insurance Coverage**									
Medicaid (%)	26	49	*	52	52	40	26	26	20
Medicare (%)	42	12	*	9	30	12	33	42	N/A
Private (%)	30	19	*	9	15	43	35	30	N/A
Self-pay (%)	19	N/A	*	26	2	5	5	1	2
Other (%)	<1	20**	*	3	1	0	1	<1	N/A

^**a**^Practices are part of the same health system and reported patient insurance coverage jointly

^**b**^These clinics had both visit-based and population outreach strategies to support CRC screening

^**c**^Includes physician (MD, DO), nurse practitioners (NP), and physician assistants (PAs)

*Unable to provide patient coverage due to internal privacy regulations

**Uninsured / No insurance

### Workflows

All primary care practices had consistent, developed workflows for colonoscopy screening and inconsistent, under-developed workflows for screening with FIT/FOBT. With respect to FIT/FOBT, all nine practices had visit-based workflows whereas only practice 2, 3 4, 5, 6 and 9 had population outreach workflows. There were four key workflow phases for FIT/FOBT provision: 1) identifying patients due for screening, 2) distributing FIT/FOBT kits to patients, 3) supporting patient reminders, and 4) updating results in EHRs and conducting patient follow up. The processes for identifying patients due for screening and distributing FITs/FOBTs varied for visit-based and population outreach workflows, whereas the processes for supporting patient reminders and conducting follow-up of FIT/FOBT results were similar under both workflows. Specific processes under each of the four key workflow phases across all nine primary care practices are summarized in Table 2 and described below.

**Table 2:** Key workflow activities and processes by primary care practice

	Primary care practice								
	Higher performing					Lower performing			
	1	2	3	4	5	6	7	8	9
**Visit-based workflow**									
Identifying patients due for CRC screening									
Protocol in place for reviewing charts before schedule clinic visits	X	X	X	X	X	X	X	X	X
Meticulous review of charts before scheduled clinic visits	X	X	X	X		X			X
Medical assistants have ability to order FIT/FOBT		X	X	X	X	X			
Distributing FIT/FOBT									
Medical assistants discuss FIT/FOBT screening with patients	X	X	X	X					
Medical assistants provide short tutorial and/or pre-label FIT/FOBT kits		X	X	X					X
Medical assistants review FIT/FOBT instructions with patients	X	X	X	X	X				
Reminders									
Standard, consistent reminders (letters and/or phone calls)		X	X	X	X		X	X	X
Documenting results and conducting follow-up									
Results from FIT/FOBT electronically uploaded into patient chart	X	X	X			X	X	X	
Medical assistants communicate normal results to patients	X	X	X	X	X	X	X	X	X
Medical assistants communicate abnormal results to patients		X			X		X		X
Nurses communicate abnormal results to patients	X	X	X	X	X			X	X
**Population outreach workflow**									
Identifying patients due for CRC screening									
Staff generate lists of patients due for screening		X	X	X	X	X			X
Distributing FIT/FOBT									
Support staff prepare and mail FIT/FOBT kits regularly		X	X	X	X	X			X
Reminders									
Standard, consistent reminders (letters and/or phone calls)		X	X	X	X				X
Documenting results and conducting follow-up									
External labs process FIT/FOBT kits and import results into patient charts		X	X			X			X
Medical assistants communicate normal results to patients		X			X				X
Medical assistants communicate abnormal results to patients		X			X				X
Nurses communicate abnormal results to patients		X			X				X

### Identifying patients due for screening

#### Visit-based workflows

Although all practices identified patients due for CRC screening, medical assistants (MAs) at practice 1 to 4, 6, and 9 meticulously reviewed patient charts before a scheduled clinic visit to identify patients due for CRC screening. For example, MAs checked records to identify previous tests completed or not completed, updated records, and met with providers to discuss appointment needs prior to meeting with the patient. In the other three practices, MAs reported having limited time to check patient charts prior to patient visits, and thus often reviewed patient charts while in the exam room with patients. Additionally, MAs at practice 2 to 6 were approved to pre-order and create orders for FIT/FOBT for clinicians to sign when the chart was routed before the patient’s appointment.

#### Population outreach workflows

Practice 2 to 6 and 9 used population outreach workflows and had staff dedicated to generating lists of patients due for CRC screening. Practices relied on health plan-funded panel managers located within the primary care clinic to identify patients due for outreach or centralized departments outside of primary care. Prior to mailing outreach, all practices (except practice 9) had primary care clinic staff review these lists to ensure patients had not completed a prior screening and/or were a good candidate for fecal testing. At practice 9, since implementation of the centralized program had recently occurred, staff did not review lists prior to mailings.

### Distributing FIT/FOBT kits to patients

#### Visit-based workflows

In all practices, FITs/FOBTs were distributed directly to patients. However, in practice 1, 2, 3, and 4, MAs initiated discussions about FIT/FOBT with patients in exam rooms and addressed CRC screening regardless of appointment type. In the other practices, providers are responsible for discussing CRC screening with patients and deciding whether to do so based on the complexity of and reason for the visit. Additionally, in practice 2, 3, 4, and 9, MAs took out kit materials to provide a short tutorial and/or pre-labeled kits to ensure accurate patient information necessary for laboratory processing and kit return, and answered patient questions. In practice 1 to 5, MA’s review FIT/FOBT return instructions with patients. In practice 6 to 9, patients were referred to the instructions that come with the FIT/FOBT kit, were instructed to simply return the kit, provided instruction only when they were identified as having trouble reading, or it was not clear what the practice did in terms of instruction review. Additionally, practices 1 to 5 reported that they provide trainings to MA staff about CRC, available CRC screening modalities, and how to talk with patients about completing and returning the FIT/FOBT.

#### Population outreach workflows

In practice 2, 3, 4, 5, 6, and 9, there were processes to prepare and mail FIT/FOBT kits to patients who were due for screening (e.g., direct mail). In practice 2, 3, and 4, this work was completed by an MA, and in practice 5, 6, and 9, either the panel manager or quality improvement nurse case manager was responsible for preparing and mailing FIT/FOBT kits.

### Supporting patient reminders

#### Visit-based and population outreach workflows

Practice 2, 3, 4, 5, 7, 8 and 9 had consistent protocols for sending patient reminders. Practice 3, 5, and 9 reminded patients during clinic visits, practice 4 and 7 called patients by telephone, and practice 6 was piloting reminder calls for patients with outstanding FIT/FOBT orders.

### Documenting FIT/FOBT results and conducting patient follow-up

#### Visit-based and population outreach workflows

All practices, except for Practice 7, provided patients with pre-stamped envelopes to enable the return of FIT/FOBT kits by mail or in person. Returned FIT/FOBT kits were processed in three different ways: by MAs or staff in their on-site laboratory (practice 4, 5, and 9), at an affiliated hospital laboratory (practice 1, 7, 8), or by an external laboratory vendor (practice 2, 3, 6). All sites had protocols for entering laboratory results into patient medical records. Practices that used on-site laboratories required staff time to process tests and then manually enter results into the patient chart. Practices that were part of a hospital system used a shared electronic health record, allowing the lab to process FIT/FOBT kits and electronically upload results into patient charts. Practices that used an external laboratory developed information technology systems to electronically upload results into the EHR or use support time for staff (MAs, panel managers, lab technicians) to manually enter faxed results into patient charts. Once kits were processed and entered into the EHR, all practices (for their visit-based workflows) tasked MA’s with calling patients with normal results. In practice 1 to 5, 8, and 9, nurses were tasked with communicating abnormal results to patients. In practice 2, 5 and 9, however, MA’s also had the ability to communicate abnormal results to patients. For population outreach workflows, practice 2, 5, and 9 tasked MA’s to communicate normal and abnormal results, whereas nurses only communicate abnormal results.

### Matrix analysis results

Table 2 shows the necessary and sufficient role of one particular workflow process vis-a-vis the outcome (i.e., higher CRC screening): Specifically, as shown in Table 2, there was perfect correspondence between the presence of the condition “MA reviews FIT/FOBT instructions with patients” and the presence of the outcome, as well as between the absence of that condition and the absence of the outcome. Additionally, in all instances where MAs discussed FIT/FOBT screening with patients, MAs also reviewed FIT/FOBT instructions with patients, indicating a direct connection between these two conditions. No other workflow processes (including combinations of processes) perfectly distinguished primary care practices with higher CRC screening from those with lower CRC screening.

## Discussion

Findings highlight the importance of standardized workflows to support higher CRC screening. In general, practice 1 to 5 had standardized processes for their visit-based and population outreach workflows for FIT/FOBT, compared to practice 6 to 9. MAs discussing and reviewing instructions for FIT/FOBT screening with patients also emerged as a difference-maker.

### Visit-based workflows

This research complements existing reports from the American Cancer Society and National Colorectal Cancer Roundtable which outline four essential steps to improving CRC screening rates for visit-based workflows in primary care clinics [36]: make recommendations, develop a screening policy, be persistent with reminders, and measure practice progress. In this study, practice 2, 3, 4, 5, and 6 had a detailed standardized course of action for identifying patients due for CRC screening that included the ability of MAs to order FITs/FOBTs for patients. Additionally, practice 1 to 4 had a process for distributing FITs/FOBTs that included MAs discussing FIT/FOBT screening and in practice 1 to 5 MA’s reviewed FIT/FOBT instructions with patients. Moreover, at practice 1 to 5, normal test results were consistently communicated by MA’s and abnormal results were communicated by nurses and MA’s. Strategies regarding communication of results were not consistent at lower performing practices.

These findings on communication and interactions from MA’s and nurses highlight the importance of team-based care in primary care. Studies have found a positive association between team-based care and strategies that promote patient engagement [37] and better health outcomes [38]. Katz et al. found that fully staffed teams are able to reconfigure roles and responsibilities and potentially develop new workflow processes [37]. Issaka and colleagues reported that having at least two members, and mostly MAs and nurses, communicate abnormal results to patients resulted in higher diagnostic colonoscopy completion [38]. Results of our matrix analysis further support the importance of engaging support staff in communication results. The specific process that corresponded perfectly to the outcome was Medical Assistants reviewing FIT/FOBT instructions with patients. Within the visit-based workflow, having defined processes for distributing FIT/FOBT kits is key to achieving higher CRC screening.

### Population outreach workflows

Although there is increasing recognition that population outreach supports timely and cost-effective care [39, 40], three practices in this study did not have population outreach workflows. A recent study by Castaneda and colleagues found that although response rates are higher with visit-based compared to population outreach interventions [41], patients reached by population outreach may be demographically distinct [42]. Thus, population outreach workflows may help increase patient outreach and thereby increase CRC screening rates. Primary care practices in national transformative initiatives, however, still use traditional staffing models rather than novel models that may be needed to operationalize comprehensive, patient-centered care [43].

### Limitations

There are several limitations of the current research. First, the study used CRC screening initiation among newly age-eligible Medicaid enrollees rather than up-to-date rates for all age-eligible patients. Although initiation rates in the highest performing quartile (32.9% to 42.9%) are lower than the national up-to-date CRC screening rate (67%) [3], these initiation rates are truly “high” considering that a) prior research by this team found that only 17% of Oregon’s newly age-eligible Medicaid enrollees completed CRC screening within one year of turning age 50 years [12] and b) CRC screening rates are significantly lower in Medicaid compared to commercially insured populations [10]. Second, practice-level rankings were based on any CRC screening modality, not just fecal testing. The focus shifted to fecal testing because of observed heterogeneity in FIT/FOBT workflows at the practice-level compared to consistent workflows for colonoscopy screening. However, multiple strategies were used to support rigor and reduce the potential for bias in this study, such as ensuring that observers were blind to practice CRC screening rates and using two observers at each observation visit [44]. Third, the study did not have access to other practice or provider characteristics, such as patient volume or provider gender and clinical training, which might influence CRC screening. Lastly, this study was conducted in the state of Oregon and thus results might not generalize to other populations. Medicaid coverage in Oregon is provided through Coordinated Care Organizations (CCO’s), networks of providers working together with local communities to provide integrated care. Although Oregon’s health care transformation may be unique, CCO’s share properties with managed care and accountable care organizations. Nonetheless, CRC screening rates remain low and there have not been systematic attempts to describe CRC screening workflows in primary care practices that provide care to Medicaid patients.

### Implications for practice and future research

Despite these limitations, findings can inform technical assistance provision and staffing required for achieving regional and national quality improvement targets for CRC screening. Along with recent data highlighting significant drops in colonoscopies and increases in delayed diagnosis of CRC due to the COVID-19 pandemic [45], findings also shed light on the need to standardize FIT/FOBT population outreach workflows. FITs/FOBTs can be performed at home and help limit in-person contact with the health care system, as well as provide screening opportunities for individuals with limited access to health care resources [45]. Future research should explore workflows associated with colonoscopy follow-up after abnormal FIT/FOBT tests, the impact of staff turnover on CRC screening rates, and how factors at the practice-level impact the effectiveness of population outreach strategies. Specifying visit-based and population outreach workflows may facilitate the implementation of evidence-based interventions into real-world, clinical settings.

## Conclusion

Key to practices with higher CRC screening was having medical assistants discuss and review FIT/FOBT screening and instructions with patients. In resource constrained settings, especially where providers have limited time to spend with patients, practices may want to focus on assigning support staff to take on the role of discussing and reviewing FIT/FOBT screening and instructions with patients. Additionally, primary care practices with higher CRC screening rates had more established visit-based and population outreach workflows to support identifying patients due for screening, FIT/FOBT distribution, reminders, and follow-up. Visit-based practices included meticulous review of medical records to identify patients due for CRC screening, training MAs to offer and review FIT/FOBT kit instructions, creating systems for patient reminders, ensuring laboratory results were integrated into the EHR, and assigning support staff to review results and follow-up with patients. Promising population outreach approaches also included having clinical team members or centralized staff generate and create lists of patients due for CRC screening, alerting patients about FIT/FOBT kit mailings, and distributing the kits. Findings from this study present important workflow processes for primary care clinics, health system leaders, and researchers working to implement or optimize existing workflows for CRC screening.

## Data Availability

The datasets generated and/or analyzed during this study are not publicly available due to the ability to identify participating practices, but are available from the corresponding author upon reasonable request.

## Abbreviations

CRC: colorectal cancer
FIT: Fecal immunochemical test
FOBT: Fecal occult blood test
USPSTF: United States Preventive Services Task Force
ACS: American Cancer Society
MAs: Medical Assistants
CCO: Coordinated Care Organization
